# Supplementary material for: Molecular Tracking of the Origin of Vesicular Stomatitis Outbreaks in 2004 and 2018, Ecuador
Source: Vet Sci. 2023 Feb 24;10(3):181. doi: 10.3390/vetsci10030181 (PMC10057135; doi:10.3390/vetsci10030181)
Supplement: Supplementary file 1 [file vetsci-10-00181-s001.zip › vetsci-2167976-supplementary.pdf]

Supplementary Table S1. Sequences obtained from GenBank NCBI-Nucleotide. The table shows the country of origin of the sample, the serotype, the accession number, the name of the strain and the reference from where it was extracted.

**Location**	**serotype**	**Accession number**	**Isolate**	**State/Province**	**Reference**
Ecuador	Indiana	EF028138.1	Ind1/Ecuador/04(5)	Loja	[16]
	New Jersey	EF028140.1	VSV_NJ/Ecuador/85	No Data	
		EF028151.1	NJ/Ecuador/04(33)	Pichincha	
		EF028150.1	VSV_NJ/Ecuador/04(20)	Guayas	
		EF028149.1	VSV_NJ/Ecuador/04(19)	Pichincha	
		EF028148.1	VSV_NJ/Ecuador/04(18)	Orellana	
		EF028147.1	VSV_NJ/Ecuador/04(11)	Los Ríos	
		EF028146.1	VSV_NJ/Ecuador/04(10)	Pichincha	
		EF028145.1	VSV_NJ/Ecuador/04(9)	Cotopaxi	
		EF028144.1	VSV_NJ/Ecuador/04(8)	Cotopaxi	
		EF028143.1	VSV_NJ/Ecuador/04(6)	Los Ríos	
		EF028142.1	VSV_NJ/Ecuador/04(3)	Cotopaxi	
		EF028141.1	VSV_NJ/Ecuador/04(2)	Napo	
Colombia	New Jersey	JX121108.1	NJ92CLB	No Data	[19]
		KU296052.1	COL/1/93		[22]
		KU296053.1	27946/COL/1998		

		KU296054.1	27775/COL/1998		
		KU296055.1	27405/COL/1998		
		KU296056.1	29453/COL/2000		
		KU296057.1	27879/COL/1998		
	Indiana	KU296058.1	28336/COL/1999		
		KU296059.1	29705/COL/2001		
United States	New Jersey	JX121110.1	NJ89GAS	Georgia	[19]
		JX121112.1	NJ95COB	Colorado	
		EF453286.1	NJ0405NME	Nuevo Mexico	[22]
		EF453235.1	NJ0905WYB	Wyoming	
		EF453237.1	NJ1005NEB3	Nebraska	
		MT094091.1	NJ0705UTB2	Utah	[23]
	Indiana	MW760857.1	Texas/18188/2019	Texas	[24]
		MW760862.1	Arizona/14629/2020	Arizona	
		MW760866.1	Nebraska/18479/2020	Nebraska	
Mexico	New Jersey	EF453267.1	NJ04CMB	Colima	[25]
		EF453306.1	NJ0803CPB4	Chiapas	
		KF772595.1	NJ1208MHB1	Michoacan	[26]
		KF772588.1	NJ1208GRB	Guerrero	
		KF772606.1	NJ1210VCB3	Veracruz	
		KF772603.1	NJ1208TBB	Tabasco	
		KF772602.1	NJ1208OAB	Oaxaca	
		KF772581.1	NJ1207CPB2	Chiapas	
		KF772593.1	NJ1208JAB5	Jalisco	
Honduras	New Jersey	JX121109.2	NJ1184HDB	No Data	[19]

		M31875.1	12/82-HD-B		[27]
		M31876.1	10/85-HD-B1		
		M31871.1	11/84-HD-B1		
		M31872.1	09/82-HD-B		
	Indiana	MK934320.1	Hon83B/L-2-83		[28]
Brazil	Indiana	JF795529.1	VSIV-386AgulhasNegrasB	Agulhas negras	[29]
		JF795530.1	VSIV-3 95Minas GeraisB	Minas Gerais	
		JF795528.1	VSIV-3 77EspinosaB	Espinoza	
		JF795527.1	VSIV-2 98Sta CatarinaB2	Santa Catarina	
		JF795525.1	VSIV-2 98ParanaE	Parana	
		JF795523.1	VSIV-2 79RiberaoE	Riberao	
		KJ746669.1	VSIV-3 2013SaoBento/ParaibaE	Sao Bento	[20]
		KJ746668.1	VSIV-3 2013Paulista/ParaibaB	Paulista	
Panama	New Jersey	M31878.1	01/85-PN-B1	No Data	[27]
		M31879.1	../60-PN-B		
		L24975.1	12/83PNB		[21]
		L24964.1	10/83PNB		
Costa Rica	New Jersey	M31874.1	10/82-CR-B		[27]
	Indiana	EF028137.1	Ind1/Costa_Rica/79		[16]
		EF028134.1	Ind1/Costa_Rica/72		

Nicaragua	New Jersey	M31877.1	07/83-NC-P		[27]
		L24971.1	11/83NCB		[21]
Guatemala		M31870.1	10/84-GM-P		[27]
Argentina	Indiana	JF795524.1	VSIV-2 86MaipuE	Buenos Aires	[29]
		JF795521.1	VSIV-2 63SaltoE	Buenos Aires	
El Salvador		EF028136.1	Ind1/El_Salvador/78	No Data	[16]
		EF028135.1	Ind1/El_Salvador/71		

Supplementary Table S2. ID of the virus samples from the 2018 outbreak, the GenBank accession number, geographic origin in Ecuador, month of isolation and serotype.

**Sample ID**	**Accession number**	**Seroype**	**Isolation Month**	**Province**	**Canton**	**Parish**
6430	ON567144	New Jersey	October	Carchi	Mira	Juan Montalvo
9084	ON567167	New Jersey	November	Carchi	Montufar	La Paz
9095	ON567169	New Jersey	November	Carchi	Espejo	San Isidro
6302	ON567142	New Jersey	October	Carchi	Mira	Juan Montalvo
3347	ON567121	New Jersey	July	Cotopaxi	Pujilí	Pujilí
5709	ON567140	New Jersey	September	Cotopaxi	Sigchos	Palo Quemado
4918	ON567127	New Jersey	August	Esmeraldas	Quinindé	Malimpia
1204	ON567116	New Jersey	March	Guayas	Balao	Balao
1499	ON567117	New Jersey	April	Guayas	Balao	Balao
6568	ON567146	New Jersey	October	Imbabura	Antonio Ante	Imbaya
6338	ON567143	New Jersey	October	Imbabura	Ibarra	Salinas
6592	ON567148	New Jersey	October	Imbabura	Urcuquí	Tumbabiro
6567	ON567145	New Jersey	October	Imbabura	Antonio Ante	Imbaya
8785	ON567156	New Jersey	October	Imbabura	Urcuquí	San Blas
8765	ON567154	New Jersey	October	Imbabura	Ibarra	Lita
8908	ON567162	New Jersey	October	Imbabura	Pimampiro	San Francisco de Sigsipamba
8825	ON567160	New Jersey	October	Imbabura	Urcuquí	San Blas

8764	ON567153	New Jersey	October	Imbabura	Pimampiro	San Francisco de Sigsipamba
8774	ON567155	New Jersey	October	Imbabura	Pimampiro	Chuga
8798	ON567157	New Jersey	October	Imbabura	Cotacachi	Sagrario
8881	ON567161	New Jersey	October	Imbabura	Pimampiro	Pimampiro
9132	ON567170	New Jersey	November	Imbabura	Urcuquí	San Blas
8992	ON567165	New Jersey	November	Imbabura	Urcuquí	Buenos Aires
8921	ON567163	New Jersey	October	Imbabura	Cotacachi	Imantag
6597	ON567149	New Jersey	October	Imbabura	Urcuquí	Pablo Salinas
2623	ON567119	New Jersey	July	Los Ríos	Montalvo	Montalvo
8789	ON567158	New Jersey	October	Morona Santiago	Morona	Sevilla Don Bosco
111	ON567115	New Jersey	January	Napo	Tena	Tena
8812	ON567159	New Jersey	October	Napo	Tena	Puerto Napo
8994	ON567166	New Jersey	November	Napo	Tena	Tena
9425	ON567175	New Jersey	November	Napo	Archidona	Cotundo
9073	ON567168	New Jersey	November	Napo	Carlos Julio Arosemena Tola	Carlos Julio Arosemena Tola
9422	ON567174	New Jersey	November	Orellana	Francisco De Orellana	Guayusa
9678	ON567177	New Jersey	December	Orellana	Francisco De Orellana	Nuevo Paraíso
8922	ON567164	New Jersey	October	Orellana	Francisco De Orellana	No Informa
9814	ON567113	Indiana	December	Orellana	Loreto	San José De Payamino
6601	ON567150	New Jersey	October	Pastaza	Pastaza	Simón Bolívar
6602	ON567151	New Jersey	October	Pastaza	Pastaza	Simón Bolívar
1785	ON567111	Indiana	April	Pichincha	Pedro Vicente Maldonado	Pedro Vicente Maldonado
4714	ON567125	New Jersey	August	Pichincha	Puerto Quito	Puerto Quito
5417	ON567138	New Jersey	September	Pichincha	Quito	Lloa
5156	ON567136	New Jersey	August	Pichincha	Pedro Vicente Maldonado	No Informa
5710	ON567141	New Jersey	September	Pichincha	Pedro Vicente Maldonado	Pedro Vicente Maldonado
9421	ON567173	New Jersey	November	Pichincha	Cayambe	Cusubamba

2482	ON567112	Indiana	June	Santo Domingo de los Tsáchilas	Santo Domingo	Valle Hermoso
3277	ON567120	New Jersey	July	Santo Domingo de los Tsáchilas	Santo Domingo	Santa María Del Toachi
4222	ON567122	New Jersey	August	Santo Domingo de los Tsáchilas	Santo Domingo	El Esfuerzo
4945	ON567129	New Jersey	August	Santo Domingo de los Tsáchilas	Santo Domingo	Alluriquín
4393	ON567123	New Jersey	August	Santo Domingo de los Tsáchilas	Santo Domingo	Las Mercedes
4498	ON567124	New Jersey	August	Santo Domingo de los Tsáchilas	Santo Domingo	Río Verde
5128	ON567134	New Jersey	August	Santo Domingo de los Tsáchilas	Santo Domingo	Las Mercedes
5126	ON567133	New Jersey	August	Santo Domingo de los Tsáchilas	Santo Domingo	Las Mercedes
5122	ON567130	New Jersey	August	Santo Domingo de los Tsáchilas	Santo Domingo	Las Mercedes
5124	ON567131	New Jersey	August	Santo Domingo de los Tsáchilas	Santo Domingo	Las Mercedes
5129	ON567135	New Jersey	August	Santo Domingo de los Tsáchilas	La Concordia	La Concordia
9639	ON567176	New Jersey	December	Santo Domingo de los Tsáchilas	Santo Domingo	Luz De América
4870	ON567126	New Jersey	August	Santo Domingo de los Tsáchilas	Santo Domingo	Alluriquín
5284	ON567137	New Jersey	August	Santo Domingo de los Tsáchilas	Santo Domingo	Puerto Limón
5125	ON567132	New Jersey	August	Santo Domingo de los Tsáchilas	Santo Domingo	Las Mercedes
5639	ON567139	New Jersey	September	Santo Domingo de los Tsáchilas	Santo Domingo	Alluriquín

4942	ON567128	New Jersey	August	Santo Domingo de los Tsáchilas	Santo Domingo	Las Mercedes
952	ON567114	New Jersey - Indiana	February	Sucumbíos	Cascales	Dorado Cascales
1992	ON567118	New Jersey	May	Zamora Chinchipe	Yantzaza	Yantzaza
9263	ON567171	New Jersey	November	Zamora Chinchipe	Chinchipe	Zumba
9356	ON567172	New Jersey - Indiana	November	Zamora Chinchipe	El Pangui	El Pangui

Suplementary Table S3. Haplotypes, and sample origin.

**Country**	**State/Province**	**Haplotype**
Colombia		Hap_1, Hap_2, Hap_3, Hap_4
Costa Rica		Hap_5
Ecuador	Carchi	Hap_30
	Cotopaxi	Hap_6, Hap_30
	Esmeraldas	Hap_32
	Guayas	Hap_6, Hap_29
	Imbabura	Hap_30, Hap_33, Hap_34, Hap_36
	Los Rios	Hap_6, Hap_8, Hap_30
	Morona Santiago	Hap_35
	Napo	Hap_7, Hap_28, Hap_30
	Orellana	Hap_9, Hap_28, Hap_38
	Pastaza	Hap_28
	Pichincha	Hap_4, Hap_6, Hap_30
	Santo Domingo de los Tsachilas	Hap_30, Hap_31, Hap_37
	Sucumbios	Hap_27
	Zamora Chinchipe	Hap_28
Guatemala		Hap_20
Honduras		Hap_11, Hap_22, Hap_25, Hap_26
Mexico	Chiapas	Hap_24
	Colima	Hap_10
	Guerrero	Hap_17
	Jalisto	Hap_19
	Michoacan	Hap_19
	Oaxaca	Hap_21
	Tabasco	Hap_21
	Veracruz	Hap_15
Nicaragua		Hap_13, Hap_16

Panama		Hap_18, Hap_23
United States of America	Colorado	Hap_14
	Georgia	Hap_12
	Nebraska	Hap_10
	Nuevo Mexico	Hap_10
	Utah	Hap_10
	Wyomin	Hap_10
